# Assessment of Metabolic Profiles in Florets of *Carthamus* Species Using Ultra-Performance Liquid Chromatography-Mass Spectrometry

**DOI:** 10.3390/metabo10110440

**Published:** 2020-10-30

**Authors:** Jiseon Kim, Awraris Derbie Assefa, Jaeeun Song, Vimalaj Mani, Soyoung Park, Seon-Kyeong Lee, Kijong Lee, Dong-Gwan Kim, Bum-Soo Hahn

**Affiliations:** 1 Department of Agricultural Biotechnology, National Institute of Agricultural Sciences, Rural Development Administration, Jeonju 54874, Korea; jiseon07100@gmail.com (J.K.); icanje@korea.kr (J.S.); vimalraj08@gmail.com (V.M.); psy0203@korea.kr (S.P.); lsk220@korea.kr (S.-K.L.); leekjong@kroea.kr (K.L.); 2 National Agrobiodiversity Center, National Institute of Agricultural Sciences, Rural Development Administration, Jeonju 54874, Korea; awraris@korea.kr; 3 Department of Bio-Industry and Bio-Resource Engineering, Sejong University, Seoul 05006, Korea; kimdg@sejong.ac.kr

**Keywords:** florets, LC-ESI-QTOF-MS, metabolites, pigment, safflower wild species

## Abstract

The genus *Carthamus* is a diverse group of plants belonging to the family Compositae. Florets of *Carthamus* species exhibit various colors, including white, yellow, orange, and red, which are related to their metabolite compositions. We aimed to investigate the metabolites accumulated in florets of three wild (*C. lanatus*, *C. palaestinus,* and *C. turkestanicus*) and one cultivated (*C. tinctorius*) species of safflower at three developmental stages. Metabolites were extracted from freeze-dried florets using 70% methanol; qualification and quantification were carried out using liquid chromatography quadrupole time-of-flight mass spectrometry in positive and negative ion modes followed by extraction of the peaks. Fifty-six metabolites, including phenylpropanoids, chalcones, isoflavonoids, flavanones, flavonols, flavones, and other primary metabolites, were identified for the first time in safflower wild species. The orange florets contained high abundances of safflomin A, anhydrosafflor yellow B, and baimaside, whereas white/cream and light-yellow pigmented florets had high abundances of 1,5-dicaffeoylquinic acid, luteolin 7-*O*-glucuronide, and apigenin 7-*O*-β-*D*-glucuronide. The principal component analysis clearly distinguished the samples based on their pigment types, indicating that color is a dominant factor dictating the identity and amount of the metabolites. Pearson correlation data based on levels of metabolites showed that orange and yellow florets were significantly correlated to each other. White and cream pigmented species were also highly correlated. Comparison between three developmental stages of safflower wild species based on their metabolite profile showed inconsistent. The findings of this study broaden the current knowledge of safflower metabolism. The wide diversity of metabolites in safflower materials also helps in efforts to improve crop quality and agronomic traits.

## 1. Introduction

The genus *Carthamus*, which probably originated in southern Asia, comprise a diverse group of plants belonging to the family Compositae. It is believed to have been cultivated in China, Egypt, India, and Iran in the era of human prehistory, and in Italy, France, and Spain during the Middle Ages [1,2]. Safflower is one of the major oil seed crops cultivated over the last five decades. *Carthamus tinctorius* L., one of the most studied major *Carthamus* species, is a branching, thistle-like herbaceous perennial broadleaved oil seed crop [3,4,5]. Other species of *Carthamus* include but are not limited to *C. alexandrines*, *C. anatolicus*, *C. creticus*, *C. dentatus*, *C. divaricatus*, *C. flavescens*, *C. glaucus*, *C. lanatus* L., *C. leucocaulos*, *C. nitidus*, *C. oxyacantha*, *C. palaestinus* L., *C. tenuis*, and *C. turkestanicus* [6,7,8,9,10,11].

Various studies have been done on the evolution of *Carthamus* species. Earlier studies indicated that the diverse nature of *Carthamus* species is accompanied by high variation in chromosome number (2n = 20, 22, 24, 44, 64; x = 10, 11, 12) [6,7,12,13]. Ashri and Knowles (1960) [7] considered *C. oxyacantha*, *C. palaestinus*, and *C. flavescens* to be true wild species closely related to *C. tinctorius*. On the other hand, these species were regarded as the biological wild species of *C. tinctorius* [14]. Other species, such as *C. creticus* and *C. turkestanicus* are treated either as distinct species or subspecies of *C. lanatus* [6,8,9,10]. Morphological, karyological, experimental hybridization, and isozyme studies indicated that *C. creticus* originated from *C. lanatus* and *C. leucocaulos*, whereas *C. turkestanicus* Popov originated from *C. lanatus* and *C. glaucus* M. Bieb subsp. Glaucus [7,9,10,15,16]. Recent studies based on phylogenetic analysis of a combined dataset [17] unweighted pair group method with arithmetic mean (UPGMA) dendrogram clustering analysis [18], nuclear DNA assay results [19], and fluorescent in situ hybridization (FISH) studies [20] showed that the cultivated *C. tinctorius* L. is closely related to, and most likely derived from, the wild species *C. palaestinus*, whereas *C. persicus* and *C. oxyacanthus* appear to be more distantly related to it. Another study based on chloroplast DNA diversity revealed that *C. oxyacantha* and *C. palaestinus* contributed their plastomes to *C. tinctorius* var. *tinctorius* and *C. tinctorius* var. *inermis*, respectively [21]. Sehgal et al. (2009) [22] reported that *C. boissieri* (2n = 20) is more likely to be a diploid progenitor of *C. lanatus* ssp. *creticus* (2n = 64), *C. lanatus* (2n = 44), *C. lanatus* ssp. *lanatus* (2n = 44), and *C. lanatus* ssp. *montanus* (2n = 44), whereas *C. glaucus* ssp. *anatolicus* (2n = 20) is a likely progenitor of *C. lanatus* ssp. t*urkestanicus* (2n = 64). FISH study results showed that *C. boissieri* is one of the genome donors for *C. lanatus* [20]. Phylogenetic analyses of the nuclear introns and additivity analysis of randomly amplified polymorphic DNA markers showed that the tetraploid *C. lanatus* is a progenitor of the hexaploid *C. turkestanicus*, and *C. glaucus* is likely the diploid progenitor of *C. turkestanicus* [11].

Petals, stamens and pistils of safflower flower exhibit various colors, including white, yellow, orange, and red [23,24]. Color is an important factor that helps as an external appearance indicator to evaluate the quality of the safflower in conformity assessments of certain specifications and quality changes due to processing, storage, and other factors. Stages of blooming affect the color of safflower florets. The most common color at early bloom is yellow, which gradually turns to red [25,26]. At the post-blooming stage, the orange, yellow, and red flowers become dark or purple [27]. Environmental conditions, such as light intensity, affect safflower color and also cause changes in color [28]. Safflower color is mostly related to its chemical components [29,30].

The various chemical constituents reported in extracts of fresh florets, oil cake, dried aerial parts, and seeds of *Carthamus* species. These include flavonoids, phenolic acids, quinochalcones, alkaloids, polyacetylene, fatty acids, sterols, triterpenes, volatile constituents, sugars, and amino acids [23,28,31,32,33,34,35,36,37,38,39,40]. The chromatic components in the petal or whole flower part of *C. tinctorius* L. reported to date include the red pigment carthamin [41], the yellow carthamin precursor precarthamin [26,42], safflower yellow B [43], safflomin A [44,45,46], safflomin C [28,47], tinctromine [48], cartormin [49], hydroxysafflor yellow A, anhydrosafflor yellow B (AHSYB) [28], hydroxycartormin, and cartormin [50]. Two quinochalcone *C*-glycosides, carthorquinosides A and B, were recently isolated from *C. tinctorius* florets [51]. Pu et al. (2019) [28] isolated flavonols and flavones from 40 diversely colored safflower floret samples. The rearranged derivatives of two flavonoid *C*-glycosides, saffloflavonesides A and B, were isolated from the florets of *C. tinctorius* [52]. Various flavonoids have been isolated from dried petals of *C. tinctorius* [53], leaves of *C. tinctorius* L. [54], and aerial parts of *C. lanatus* [38,55]. Fresh florets of *C. tinctorius* L. were reported to include derivatives of kaempferols and quercetins [23]. The petals of *C. tinctorius* L. are a rich source of volatile oils [1]. The most common fatty acids extracted from safflower include oleic, linoleic, palmitic, and stearic acids [56,57,58]. Aromatic glucosides, alkaloids, *p*-coumaric acid and its derivatives, coumaroylspermidine analogs, and various types of glucopyranosides have also been isolated from *C. tinctorius* [53,59,60,61] Other compounds such as oxygenated bisabolanefucosides, asperulosides, stigmasterol 3-*O*-β-*D*-glucoside, and sitosterol 3-*O*-β-*D*-glucoside were isolated from aerial parts of *C. lanatus* [39]. Flavonoids and quinochalcones are considered the main and active constituents of safflower plant extracts. *Carthamus tinctorius* L. is the most investigated species in terms of its chemical constituents.

Safflower is an important crop with various applications. The florets and seeds of safflower have been used in various pharmaceutical and industrial applications, including producing herbal medicines and food colorants, as a natural red dye, and as a source of vegetable and industrial oils [3,62,63,64,65]. Traditionally, safflower florets have been used in China, Korea, Japan, and other countries for the treatment of various cardiovascular-related diseases, inflammatory diseases, osteoporosis, and gynecological disorders [66,67]. Modern clinical and epidemiological experiments indicate that safflower helps in dilating coronary arteries, improving myocardial ischemia, and regulating the immune system; it also possesses anticoagulative, antithrombotic, and antioxidative properties [67,68]. The chromatic component of orange and red safflower, hydroxysafflor yellow A, was reported to reduce blood pressure and heart rate [65]. Whole extracts, fractions, and constituents of *C. lanatus* that contained quercetin and luteolin glucosides as the main constituents increased the amount of glutathione antioxidant in human endothelial EA.hy926 cells [69] and inhibited cell proliferation [70]. In other studies, dichloromethane, water, and methanol extracts of *C. lanatus* L. exhibited significant anti-inflammatory activity in induced human neutrophils and rats [71,72], as well as noticeable antibacterial activity and cytotoxicity [73].

Although the identification and quantification of secondary and primary metabolites have been reported for the cultivated safflower species *C. tinctorius*, safflower wild species have received limited attention. To the best of our knowledge, there are no reports concerning the metabolites of the safflower wild species *C. lanatus*, *C. palaestinus*, and *C. turkestanicus*. Profiling the metabolites in *Carthamus* spp. florets expands our knowledge of bioactive components and is important for quality control improvement. In this paper, we evaluated differences in the quality and quantity of metabolites between three wild and one cultivated safflower species. First, 56 metabolites were identified from safflower florets sampled at the early, middle, and late maturity stages using liquid chromatography quadrupole time-of-flight mass spectrometry (LC-ESI-QTOF-MS). Second, the metabolite variations among different species were assessed using multivariate data analysis. Third, the absolute concentrations of metabolites that differed significantly among species were examined. This study broadens our knowledge of flowering behavior so as to identify physiological process indicators and highlights the value of safflower as a source of natural colorants, food additives, and nutraceuticals.

## 2. Results

### 2.1. Floret Colors and Leaf Shapes of Safflower Species

**Figure 1:**
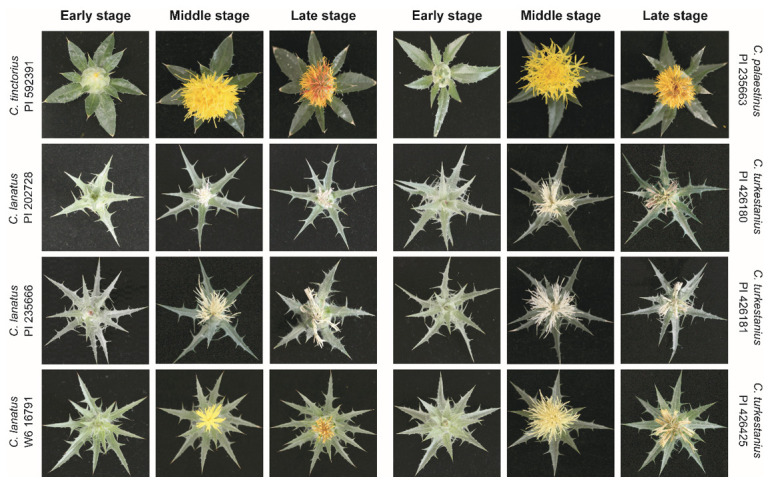
Phenotypes of wild safflowers and cultivars. Safflower materials were obtained from the USDA (U.S. Department of Agriculture) National Plant Germplasm System via the Germplasm Resource Information Network. *C*. *tinctorius* and *C*. *palaestinus* florets were orange at the middle stage and became red/orange as development proceeded. Florets of W6 16791 and PI 426425 were yellow at the middle stage, and the pistils became orange as development proceeded. All other materials were white/cream at the middle stage.

Florets of three wild (*C*. *palaestinus*, *C*. *lanatus*, and *C*. *turkestanicus*) and one cultivated (*C*. *tinctorius*) safflower species at three different growth stages were used in this study; photographs are presented in Figure 1. Floret samples were collected at three different developmental stages as described previously [25]: (1) before the beginning of flowering, when the upper portion of the florets about to emerge through the bracts (early stage); (2) at the stage when the flowering is considered complete (more than 90% of florets open); and (3) at the late stage of flowering when the capitulum begins to expand and the seeds are about to start developing. At the complete flowering stage (middle stage), the florets of the studied genotypes were yellow, light-yellow, and white/cream. At later stages, the yellow and light-yellow florets started to change color to orange or red-orange. The florets of *C*. *tinctorius* and *C*. *palaestinus* are orange at the middle stage and orange/red at the late stage. W6 16791 (*C*. *lanatus*) and PI 426425 (*C*. *turkestanicus*) are yellow and light-yellow at the middle stage of growth, respectively. The former changed color to yellow/red, whereas little color change was observed for the latter. The petals of PI 235666 (*C*. *lanatus*), PI 426180 (*C*. *turkestanicus*), and PI 426181 (*C*. *turkestanicus*) were white at the middle stage of development. The pistils of the other flowers were either yellow or yellowish except the pistils of PI 202728 (*C*. *lanatus*), which were white at the middle stage. The involucral bracts of the main capitula and leaf margins of *C*. *tinctorius* and *C*. *palaestinus* exhibited short spines, whereas those of all other species had long and sharp spines. All species generally had lanceolate leaf shapes, but the leaves of *C*. *tinctorius* and *C*. *palaestinus* were relatively wider with acuminate apices, whereas those of the other species had attenuate leaf apices. Head sizes are described as small, intermediate, and large. The florets of *C*. *tinctorius* and *C*. *palaestinus* had large and intermediate head sizes, respectively. Those of the other species had small head sizes.

### 2.2. Metabolites of Safflower Species

A total of 56 metabolites, including 19 flavonols, 9 flavones, 9 phenylpropanoids, 4 flavanones, 4 isoflavones, 5 primary metabolites, 2 chalcones, 2 anthraquinones, esculetin, and salicylic acid, were identified in safflower florets using LC-ESI-QTOF-MS. The relative peak areas of the identified metabolites are presented in Table S1. Flavones (apigenin 7-*O*-β-*D*-glucuronide, luteolin 7-*O*-glucuronide, and luteolin 7-*O*-β-*D*-glucoside), flavonols (astragalin, kaempferol, quercetin, isoquercetin, kaempferol 3-*O*-β-*D*-glucosylgalactoside, and kaempferol 7-*O*-β-*D*-glucopyranoside), phenylpropanoids (caffeic acid, caffeoylquinic acid, and quinic acid), safflomin A, and aconitic acid were detected in all samples. The chromaticity-related components safflomin A and AHSYB were among the dominant metabolites in the orange florets of *C*. *tinctorius* and *C*. *palaestinus*. Luteolin 7-*O*-glucuronide comprised the highest portion of the total content of metabolites in the white and creamy florets of *C*. *lanatus* and *C*. *turkestanicu*s. Instead of quinochalcones, isoflavonoids were the dominant compounds in white and creamy florets. Malonylgenistin, pratensein, and 2’-hydroxygenistein were detected in *C*. *turkestanicus* and *C*. *lanatu*s, whereas 6-hydroxydaidzein was only detected in *C*. *lanatus* and the early-stage florets of *C*. *palaestinus*. Most of the flavones and flavonols were detected in most samples with some exceptions. Lesser quantities of 3’-*O*-methylluteolin, apigenin, 3-*O*-methylquercetin, myricitrin, and quercetin 3-*O*-(6-*O*-malonyl-β-*D*-glucoside) were detected in orange and yellow florets compared with others. Phenylpropanoids were distributed throughout the samples with 1,5-dicaffeoylquinic acid, caffeoylquinic acid, and quinic acid contributing highly to the overall content of phenylpropanoids. High interspecies variation was observed in the levels of some of the metabolites. Whereas *p*-coumaric acid dominated in *C*. *tinctorius* and *C*. *palaestinus* florets, 1,5-dicaffeoylquinic acid was only detected in *C*. *turkestanicus* and *C*. *lanatus*.

Correlation analysis can be used to substantiate the relationship between biochemically related properties in plant samples. To examine the detailed relationships among floret samples, Pearson correlation analysis was performed using the relative peak areas of the 56 studied compounds (Table 1). Two *C*. *lanatus* individuals, PI 235666 and PI 202728, which had creamy and white florets, respectively, exhibited similar metabolite profiles and were strongly correlated with each other (R^2^ = 0.647–0.976). However, the yellow *C*. *lanatus* (W6 16791) was not significantly correlated with PI 235666 or PI 202728 despite being the same species. W6 16791, rather, was highly correlated with *C*. *tinctorius* and *C*. *palaestinus* and moderately correlated with yellow *C*. *turkestanicus* (PI 426425). A similar correlation trend was observed between the cream pigmented *C*. *lanatus* and *C*. *turkestanicus*, suggesting that, rather than genotype, color is a strong predictor of the quality and quantity of metabolites in safflower florets. All *C*. *turkestanicus* individuals exhibited very high similarity in metabolite profile and were significantly correlated with each other and with the creamy and white *C*. *lanatus* individuals. The yellow variant of *C*. *turkestanicus* (PI 426425) was moderately correlated with the orange *C*. *tinctorius* and *C*. *palaestinus* and the yellow *C*. *lanatus* (W6 16791), confirming greater resemblance to the creamy and white variants. Hierarchical clustering analysis (results not shown) of the florets at the middle stage of flowering identified two major clusters. One group contained individuals of *C*. *tinctorius* and *C*. *palaestinus* as well as the yellow *C*. *lanatus* (W6 16791). All remaining species were clustered in the other group.

**Figure 2:**
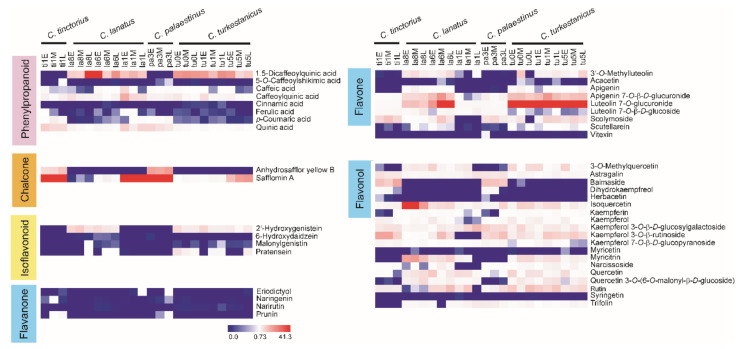
Heat maps comparing the levels of phenylpropanoids, chalcones, and flavonoids in safflower wild species. Relative peak areas were normalized to construct a comparative heat map. The abscissa at top displays the names of the samples, and the ordinate at right displays the names of the metabolites. The deeper the red color, the higher the peak area of the metabolites; the deeper the blue color, the lower the peak area of the metabolites. Sample name abbreviations: the first two letters indicate the first two letters of the species name, the number indicates the last digit of the PI number, and the letters E, M, and L indicate early, middle, and late stages of development, respectively. For example, “ti1E” indicates the sample PI 592391 (*C*. *tinctorius*) at the early stage of development.

A heat map analysis (Figure 2) based on the relative peak areas of the metabolites was performed to examine variation in metabolite synthesis among species and floret developmental stages. The orange florets of *C*. *tinctorius* and *C*. *palaestinus* contained high levels of safflomin A, AHSYB, and baimaside, whereas the other florets contained high levels of 1, 5-dicaffeoylquinic acid, luteolin 7-*O*-glucuronide, and apigenin 7-*O*-β-*D*-glucuronide. Other flavonoids, such as dihydrokaempferol, eriodictoyl, naringenin, and prunin were either not abundant or identified at trace levels in white/creamy and light-yellow florets. Some other metabolites that are involved in flavone and flavonol biosynthesis, including syringetin, vitexin, acacetin, herbacetin, narirutin, and myricetin, were not detected in most of the materials. Compared with the yellow and orange florets, 2’-hydroxygenistein was more abundant in white/creamy and light-yellow florets, whereas the other isoflavonoid, 6-hydroxydaidzein, was much less abundant and not detected in most of the safflower species.

### 2.3. Classification of Safflower Species Based on Their Characteristic Chemical Components Using Principal Component Analysis (PCA) and Orthogonal Partial Least Squares Discriminant Analysis (OPLS-DA)

**Figure 3:**
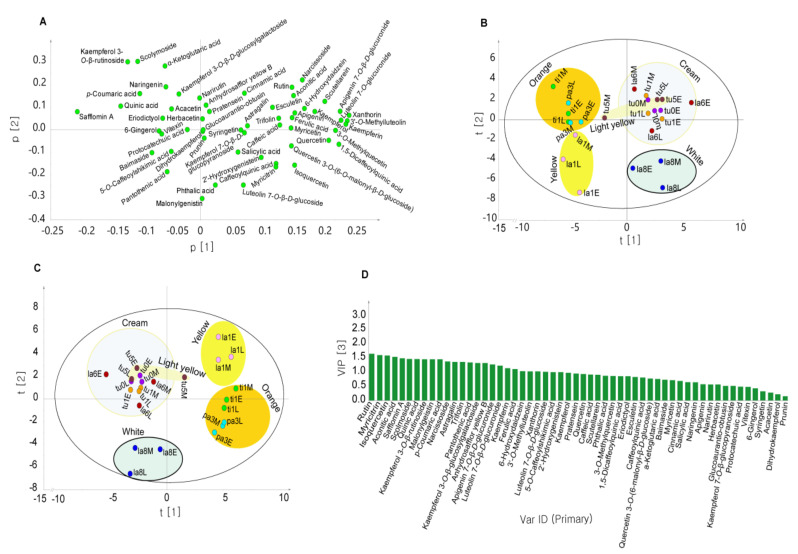
Loading (**A**) and score (**B**) plots of principal components 1 and 2 from the principal component analysis (PCA) and orthogonal partial least squares discriminant analysis (OPLS-DA) (**C**) and variable importance in projection (VIP) scores associated with metabolites (**D**) of wild and cultivated safflower species. Sample name abbreviations: the first two letters indicate the first two letters of the species name, the number indicates the last digit of the PI number, and the letters E, M, and L indicate early, middle, and late stages of cultivation, respectively. For example, “ti1E” indicates the sample PI 592391 (*C*. *tinctorius*) at the early stage of development.

PCA and OPLS-DA scatter plots representing the safflower samples and loading plots of their 56 constituents are presented in Figure 3. In the PCA plot, the orange *C*. *tinctorius* and *C*. *palaestinus*, yellow *C*. *lanatus*, and white *C*. *lanatus* (PI 202728) were clearly separated, with sample points located at the top left, bottom left, and bottom right quadrant, respectively. All three individuals of *C*. *turkestanicus* and one *C*. *lanatus* (PI 235666) individual formed a loose group at the top right PCA plot quadrant, but the middle stage florets of light-yellow *C*. *turkestanicus* (PI 426425) were excluded. The pistils of these safflower wild species were yellow except for PI 202728. Factor loadings in the first component revealed that the compounds with the highest contributions were kaempferin, luteolin 7-*O*-glucuronide, apigenin 7-*O*-β-*D*-glucuronide, 1,5-dicaffeoylquinic acid, xanthorin, 2′-hydroxygenistein, quercetin, 3-*O*-methylquercetin, scutellarein, 3′-*O*-methylluteolin, quercetin 3-*O*-(6-*O*-malonyl-β-*D*-glucoside), narcissoside, apigenin, myricitrin, and kaempferol, with eigenvectors ranging between 0.22 and 0.15, which indicated that they were present at higher levels mainly in the early-stage florets of PI 235666 (*C*. *lanatus*) and all individuals of *C*. *turkestanicus*. Safflomin A, baimaside, AHSYB, glucoaurantio-obtusin, and dihydrokaempferol contributed the least to component 1 (eigenvector values between −0.21 and −0.15), further confirming that the orange and yellow florets (all grouped in the left [negative] side of the PCA plot) contained higher levels of these components. Other constituents, including rutin, *p*-coumaric acid, aconitic acid, kaempferol 3-*O*-β-*D*-glucosylgalactoside, scolymoside, and kaempferol 3-*O*-β-rutinoside, contributed highly to component 2 with eigenvectors ranging from 0.21 to 0.34, whereas pantothenic acid, caffeoylquinic acid, and syringetin had negative eigenvector values of −0.25, −0.17, and −0.15, respectively. OPLS-DA was performed to examine the distribution in the safflower species and highlight the distinguishing metabolites. The metabolites with statistically significant relevance for explaining the differences among species were identified. Twenty-four metabolites had variable importance in projection (VIP) scores greater than or equal to 1. Rutin, myricitrin, isoquercetin, and aconitic acid had the highest contributions in decreasing order with VIP scores greater than 1.5. The score plot of the OPLS-DA (Figure 3c) clearly revealed the separation of most safflower species based on floret color. Each of the orange, yellow, white, and cream colored species essentially appeared in different quadrants of the OPLS-DA plot except for PI 426425 (*C*. *turkestanicus*) at the early and late stages of development, which were grouped together with creamy-floret species.

### 2.4. Quantification of Metabolites with High VIP Scores

**Figure 4:**
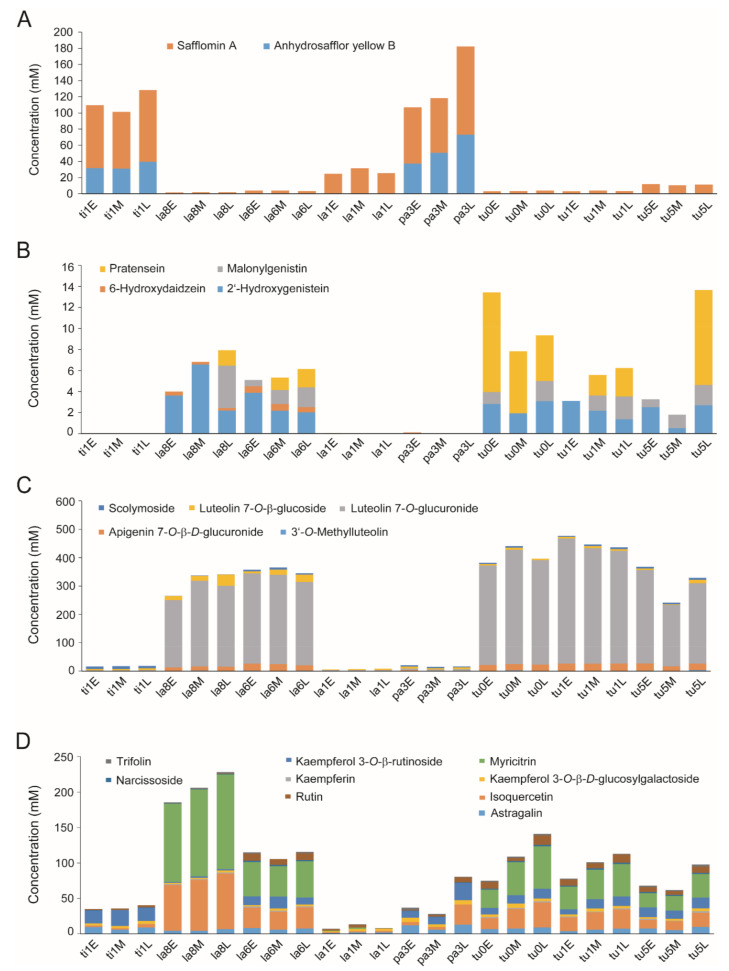
Cumulative distribution of chalcones (**A**), isoflavonoids (**B**), flavones (**C**), and flavonols (**D**) in wild safflower florets at different developmental stages. Sample name abbreviations: the first two letters indicate the first two letters of the species name, the number indicates the last digit of the PI number, and the letters E, M, and L indicate early, middle, and late stages of development, respectively. For example, “ti1E” indicates the sample PI 592391 (*C*. *tinctorius*) at the early stage of development.

The contents of metabolites that significantly contributed to discriminating among safflower species are presented in Figure 4 and Table S2. The chromaticity-related components safflomin A and AHSYB were among the dominant metabolites in *C*. *tinctorius* and *C*. *palaestinus*. Safflomin A was detected in all samples, with contents ranging from 1.64 to 109.14 mM, whereas AHSYB was only found in *C*. *tinctorius* and *C*. *palaestinus* and varied between 31.17 and 73.13 mM. Isoflavonoids and flavones, unlike quinochalcones, accumulated at higher levels in non-orange and non-yellow florets. Pratensein and 2’-hydroxygenistein dominated in *C*. *turkestanicus* and *C*. *lanatus*, respectively. Most of the isoflavonoids were not detected in *C*. *tinctorius*, *C*. *palaestinus*, and yellow-pigmented *C*. *lanatus* (W6 16791), and 6-hydroxydaidzein was detected only in *C*. *lanatus* (PI 202728 and PI 235666). A similar trend was observed in flavones with luteolin 7-*O*-glucuronide ranging from 216.08 to 440.72 mM in *C*. *turkestanicus* and two *C*. *lanatus* (PI 202728 and PI 235666) individuals. The flavonols were distributed in all species; *C*. *lanatus* (PI 202728) with white florets accumulated the largest amount, whereas *C*. *lanatus* (W6 16791) with yellow florets accumulated the lowest. Myricitrin and isoquercetin, whose contents ranged from 0.00 to 133.43 mM and 1.16 to 78.42 mM, respectively, contributed the most to the overall levels of flavonols with high VIP scores. Myricitrin was not detected in orange florets of *C*. *tinctorius* and *C*. *palaestinus*. Phenylpropanoids were distributed throughout all species with 1,5-dicaffeoylquinic acid, ferulic acid, and *p*-coumaric acid predominating. However, the identities of the dominant compounds varied among species. Whereas *p*-coumaric acid was dominant in *C*. *tinctorius* and *C*. *palaestinus*, 1,5-dicaffeoylquinic acid was only detected in *C*. *turkestanicus* and *C*. *lanatus* (Table S2).

## 3. Discussion

Various morphological descriptions of safflower have been published [74,75]. Floret color is an important phenotypic trait that indicates the chemical characteristics of safflower. Safflower florets exhibit several colors ranging from white to yellow/red/orange depending on the variety, genotype, and developmental stage [4]. Yellow is the predominant petal color in many *Carthamus* spp. [76]. Previously collected data accessions of *C. tinctorius* variants revealed that white, pale yellow, yellow orange, orange, orange red, and red pigments exist, as well as both spiny and spineless and small-, medium-, and large-headed florets [77,78]. Data on the phenotypes of the safflower wild species *C. lanatus, C. palaestinus*, and *C. turkestanicus* in the literature are lacking.

In this study, the metabolic profiles of wild and cultivated safflower species were studied using LC-ESI-QTOF-MS. Flavonols absorb wavelengths of approximately 340 nm due to the flavonol aglycone. Flavonoids have ether, ester, and C4–C8 bonds that can be easily cleaved in mass analysis. The retention times of the metabolites, comparison with commercial standards, comparison of mass fragmentation patterns with those in databases (in-house chemical libraries and public libraries), and the literature simultaneously assist in the chemical assignment of aglycone structures and their derivatives. Flavonol and flavone glycosides in safflower mainly consist of combinations of aglycones, glucuronidation, glycosylation, and sugar groups. Flavonoid fragmentation is characterized by the elimination of sugar residues and the formation of aglycone product ions [79,80,81]. In this study, luteolin, apigenin, kaempferol, quercetin, myricetin, and naringenin were prevalent aglycones of safflower florets. Quinochalcones occur as monomeric or dimeric *C*-glucosides and display a prominent band in their ultraviolet–visible spectra at approximately 403 nm, which helps to differentiate them from other flavonoids [66]. Safflomin A and AHSYB quinochalcones were identified in this study. The specific fragmentation patterns of quinochalcones from *C. tinctorius* have been previously described [50,82] and reviewed [66].

Metabolite profiling was combined with chemometrics with the goal of identifying specific constituents based on species and color of safflower florets. PCA can reveal intrinsic similarities and differences in metabolite abundance in a given sample collection. In this study, PCA was performed to classify 24 floret samples (three wild and one cultivated safflower species at three different developmental stages) based on the relative peak areas of 56 identified chemical components. OPLS-DA was conducted to validate the classification. The PCA and OPLS-DA results revealed that the samples tended to form groups based on floret color. The VIP score represents the contribution of each constituent to the OPLS-DA model. The larger the VIP score, the greater the contribution. Usually, components with VIP scores greater than 1 are considered more important in distinguishing samples. The chemical components with higher VIP scores were closely related to color characteristics.

Accumulation of metabolites in safflower florets is highly influenced by various factors, including harvest time and/or flower development [27,83,84], color [28,29,30], genotype/cultivar [23,84,85], and drought stress [86]. Safflowers with different colors exhibit a wide variety in the quality and quantity of their chemical constituents. For example, safflomin A levels decrease as safflower florets become less red and darker [29]. In another study, orange and white safflower florets contained high levels of safflomin A and kaempferol-3-*O*-β-*D*-glucoside, respectively [30]. Strong associations between various chemical components and color were reported in safflower [28] and other food samples [87]. Harvest time affected the levels of red and yellow pigments in *C. tinctorius*, with levels of yellow components peaking at the beginning of flowering and decreasing during flower development, whereas those of the red components increased [27,84]. This is in concordance with safflower florets reddening during development. In the OPLS-DA plot (Figure 3), safflomin A and quinic acid are located in the bottom-right quadrant, similar to the orange samples, whereas scolymoside and kaempferol 3-*O*-β-rutinoside are found in the same location as the yellow samples. This suggests that these compounds contribute to the corresponding orangeness and yellowness of the florets. Pu et al. (2019) [28] reported that safflomin A, AHSYB, and safflomin C made safflower florets brighter, and more red, yellow, and orange-yellow, whereas 6-hydroxykaempferol-3-*O*-β-*D*-glucoside and kaempferol-3-*O*-rutinoside made safflower florets more orange-yellow. In our study, floret color seems to be a very important indicator of metabolite accumulation. The two orange cultivated (*C. tinctorius*) and wild (*C. palaestinus*) species of safflower had similar metabolite profiles despite being distinct species. This could be partly due to their similar floret color and the genetic relationship between the two species as indicated by the phylogenetic studies described in the introduction section. Choice of extraction solvent dictates the identity and quantity of metabolites that could be recovered from plant sources. Methanol is a commonly used solvent for extraction of hydrophilic polyphenols [88,89]. Methanol has been used to extract phenolic compounds from safflower plant [28,85,90]. This study was mainly focused on phenylpropanoids and flavonoids.

Absolute quantification of the metabolites that had high VIP scores and significant contributions for discrimination analysis was performed. Although the identity and quantity of some metabolites varied among floret developmental stages, the pattern of variation was inconsistent and, hence, inconclusive. However, during the floret developmental stages in *C. tinctorius*, changes in polyphenol, flavonoid, and proanthocyanidin contents have been reported, with the levels peaking at the middle stage [83]. A comparison of the levels of metabolites quantified in our study revealed that orange flowers had higher levels of quinochalcones, whereas white and creamy ones accumulated higher levels of isoflavonoids, flavones, and flavonols. Salem et al. (2011) [83] found that the level of total flavonoids and total phenolic compounds increased in the following order: yellow < red < orange flowers.

**Figure 5:**
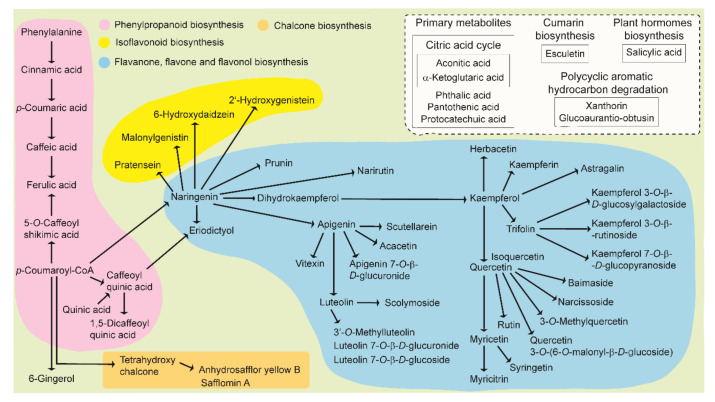
Simplified integrated metabolic pathways of phenylpropanoid, chalcone, isoflavonoid, flavanone, flavone, and flavonol biosynthesis in the florets of wild and cultivated safflower species.

The chemical components of safflower identified in this study are involved in various metabolic pathways. The main biosynthetic metabolic pathways involve phenylpropanoids, chalcone isoflavonoids, flavanones, flavones, and flavonols. Simplified metabolic pathways that include phenylpropanoids and flavonoids in safflower florets are presented in Figure 5. The phenylpropanoid and polyketide pathways are responsible for the biosynthesis of flavonoids. The former is derived from phenylalanine and tyrosine and is responsible for the formation of *p*-coumaroyl-CoA, whereas the latter is responsible for the elongation of the C2 chain using malonyl-CoA as the condensing unit. The first enzyme responsible for the biosynthesis of flavonoids is chalcone synthase. The formation of naringenin chalcone is catalyzed by chalcone synthase in the polyketide pathway from *p*-coumaroyl-CoA and malonyl-CoA [90]. Naringenin chalcone undergoes stereospecific cyclization to form a flavanone, naringenin, with the help of the enzyme chalcone isomerase. Naringenin plays a central role in the metabolic pathway for the formation of other flavanones, isoflavonoids, flavones, and flavonols. Chalcone isomerase also converts tetrahydroxychalcone into naringenin. Flavanone 3-hydroxylase catalyzes the oxygenation of naringenin at the 3-position to form dihydrokaempferol (aromadendrin), which is subsequently converted to kaempferol, which then is converted to quercetin. Shikimate/quinate hydroxycinnamoyltransferase genes convert *p*-coumaroyl-CoA into caffeoyl-CoA, which is further converted to eriodictoyl or 1,5-dicaffeoylquinic acid. Flavonoid scaffolds also undergo several tailoring reactions, such as glycosylation, methylation, and acylation, resulting in the formation of diverse metabolites with different physicochemical and biological properties, catalyzed by flavonoid glycosyltransferase, flavonoid methyltransferase, and flavonoid acyltransferase, respectively.

## 4. Materials and Methods

### 4.1. Plant Samples and Chemicals

Three wild *Carthamus* species (*C*. *lanatus*, *C*. *palaestinus*, and *C*. *turkestanicus*) and a cultivated species, *C*. *tinctoriu*s, were obtained from the USDA National Plant Germplasm System via the Germplasm Resource Information Network and planted in a greenhouse maintained at 18–25 °C located at the National Institute of Agricultural Sciences, Jeonju, Korea. Sample information and some phenotypic characters are presented in Table 2. Flowers from the plants were collected by handpicking at three developmental stages (early, middle, and late stages). Since the stages of flower development were not morphologically the same for the different species, the florets were collected at different time. Early stage samples were collected before the onset of flowering; the middle stage samples at the stage when the flowering is considered complete (more than 90% of florets open); and the late stage samples were collected when the capitulum begins to expand and the seeds are about to start developing. The flowers of each plant were continuously monitored and quickly collected at the required stage of development. Sample collection was initiated at approximately 12 weeks after seed planting. At the early stage, flower heads were removed and washed with distilled water, after which excess water was removed using filter paper. Flowers at the middle and late stages were directly collected by handpicking from flower heads. Samples were snap-frozen using liquid nitrogen, then freeze-dried and stored at –80 °C until further processing.

All reagents were LC/MS-grade. Methanol, acetonitrile (ACN), and water were purchased from Merck (Darmstadt, Germany). Formic acid was obtained from Sigma-Aldrich (St. Louis, MO, USA). Standards of quinic acid, aconitic acid, pantothenic acid, 5-*O*-caffeoylshikimic acid, kaempferol 3-*O*-β-*D*-glucosylgalactoside, rutin, *p*-coumaric acid, myricitrin, scolymoside, isoquercetin, luteolin 7-*O*-β-*D*-glucoside, safflomin A, ferulic acid, narcissoside, kaempferol 3-*O*-β-rutinoside, astragalin, luteolin 7-*O*-glucuronide, trifolin, 1,5-dicaffeoylquinic acid, 6-hydroxydaidzein, apigenin 7-*O*-β-*D*-glucuronide, kaempferin, malonylgenistin, AHSYB, 2′-hydroxygenistein, xanthorin, pratensein, and 3′-*O*-methylluteolin were purchased from ChemFaces (Wuhan, China).

### 4.2. Sample Preparation and Extraction

For metabolite extraction, all samples were freeze-dried, and 25 mg of each sample was weighed and transferred to a cryotube (2.0 mL) containing zirconia beads (YTZ-5; 5-4060-13; 5-mm diameter; AS ONE, Osaka, Japan). Into each cryotube, 1 mL of 70% methanol was added. The sample solutions were homogenized using a beads shocker operating at 25 Hz for 2 min (at 4 °C) five times, followed by centrifugation at 15,000× *g* for 5 min (at 4 °C). Then, the upper aqueous layer was carefully removed (~800 μL) and filtered using prewashed filters (0.2 µm, GHP, 13 mm; Pall, Port Washington, NY, USA). The filtrates containing the metabolites were diluted and mixed with an internal standard (2.5 µm, 7-hydroxy-5-methylflavone) at a 1:1 ratio. Then, the extracts were transferred into a 2-mL amber vial and injected into the LC-ESI-QTOF-MS system. The samples were analyzed in biological triplicates.

### 4.3. LC-ESI-QTOF-MS Analysis of Metabolites

Metabolite qualification and quantification was carried out using a Shimadzu liquid chromatography system (Nexera X2 UHPLC; Shimadzu, Kyoto, Japan) equipped with a quadrupole time-of-flight mass spectrometer (AB Sciex, Ontario, CA, USA) in positive and negative electrospray ionization (ESI) modes, followed by peak extraction using automatic integration software (MasterView v1.1; AB Sciex). Metabolites were analyzed under the following conditions. The LC-ESI-QTOF-MS system was controlled by Shimadzu software (Lab Solutions v5.73; Nexera X2 UHPLC; Shimadzu) for liquid chromatography, and a reversed-phase column (Shim-pack GIS-ODS-I column, 3 µm, 3.0 × 100 mm; Shimadzu) was used. The run solution was 0.1% formic acid in ACN, which was also used for rinsing. The column oven temperature was maintained at 40 *°*C, and the mobile phase was composed of 0.1% formic acid in water (eluent A) and 0.1% formic acid in ACN (eluent B). The elution conditions were as follows: 0–1 min, 10% of eluent B; 1–25 min, 10–100% of eluent B; 25–40 min, 100% of eluent B; and 40–41 min, 10% of eluent B. The flow rate and injection volume were maintained at 0.5 mL/min and 5 μL, respectively. Mass spectrometry conditions were maintained as follows: nebulizing gas, 50 psi; heating gas, 50 psi; curtain gas, 25 psi; temperature, 550 *°*C; ion spray voltage, floating between 4.5 and 5.5 kV; fragmentation, 35 collision energy; and 15 collision energy spread, TOF/MS and TOF/MS/MS scan ranges of 50–1500 and 50–1500 m/z, respectively. Samples were diluted two-fold and five-fold for the positive and negative ionization modes, respectively, to improve analysis quality.

### 4.4. Qualitative Analysis and Data Processing 

Fifty-six metabolites were identified based on comparisons of retention times and mass fragmentation patterns with commercial standards, previous reports, a mass spectrometry database (The Accurate Mass Metabolite Spectral Library; AB Sciex), and an in-house library. Peaks were automatically extracted using MasterView integration software to obtain various peak information, including m/z value, migration time (MT), and peak area. All signal peaks potentially corresponding to authentic compounds were extracted, whereas others corresponding to isotopomers, adduct ions, and other product ions of known metabolites were excluded. The MTs of extracted peaks were normalized using the MTs of the internal standards, followed by peak alignment according to the m/z values and normalized MT values. Finally, peak areas were normalized against that of the internal standard (7-hydroxy-5-methylflavone). The resultant relative peak area values were further normalized by sample amount. Annotation tables were constructed from LC-ESI-QTOF-MS analysis of authentic standards and aligned with the datasets based on similar m/z and normalized MT values. The peak detection limit was determined according to the signal-to-noise ratio, which was 20. The relative peak area was calculated as follows:

$$\mathrm{Relative}\;\mathrm{Peak}\;\mathrm{Area}=\;\frac{\mathrm{Metabolite}\;\mathrm{Peak}\;\mathrm{Area}\;}{\mathrm{Internal}\;\mathrm{Standard}\;\mathrm{Peak}\;\mathrm{Area}\;}$$

### 4.5. Quantification of Metabolites and Statistical Analysis

Absolute quantification of 27 metabolites was performed. The concentrations of the metabolites were calculated using linear regression equations derived from the calibration curves of the corresponding commercial standards. Results are presented as the mean ± standard deviation of triplicate experiments. Pearson correlation analysis was performed using SPSS v17.0 statistical software (SPSS Inc., Chicago, IL, USA). PCA and OPLS-DA were performed using SIMCA v13.0.3 software (Umetrics, Umea, Sweden). Quantitative expression of the metabolites was normalized using the preprocessCore package in Bioconductor software [91], and heat maps were generated using MeV v4.9.0 software [92].

## 5. Conclusions

Wide diversity in quality and quantity of metabolites among safflower species were explored. A total 56 metabolites were identified and absolute quantification of 27 significantly differential metabolites was performed. The cultivated safflower species, *C. tinctorius* and the wild safflower species, *C*. *palaestinus* showed a strong resemblance to each other in terms of the identity and amount of metabolites. The orange colored florets showed high abundance of safflomin A, anhydrosafflor yellow B, and baimaside while white/whitish and light yellow pigmented florets had high abundance of 1, 5-dicaffeoylquinic acid, luteolin 7-*O*-glucuronide, and apigenin 7-*O*-β-*D*-glucuronide. Data were analyzed using multivariate statistical methods, PCA and OPLS-DA, and heat maps. The results demonstrated that a clear separation of the samples based on their color, indicating that color is a dominant factor dictating the identity and quantity of the metabolites.

## Figures and Tables

**Table 1:** Pearson correlation coefficients among safflower wild species at different developmental stages based on metabolite levels.

Species		*C. tinctorius* PI 592391			*C. lanatus* PI 202728			*C. lanatus* PI 235666			*C. lanatus* W6 16791			*C. palaestinus* PI 235663			*C. turkestanicus* PI 426180			*C. turkestanicus* PI 426181				*C. turkestanicus* PI 426425	
		E	M	L	E	M	L	E	M	L	E	M	L	E	M	L	E	M	L	E	M	L	E		M
*C. tinctorius* PI 592391	M	0.998**																							
	L	0.998 **	0.996 **																						
*C. lanatus* PI 202728	E	−0.077	−0.083	−0.076								Key													
	M	−0.088	−0.092	−0.086	0.976 **							−1	−0.75	−0.5	−0.25	0	0.25	0.5	0.75	1					
	L	−0.081	−0.087	−0.079	0.940 **	0.914 **																			
*C. lanatus* PI 235666	E	0.008	0.015	0.002	0.710 **	0.742 **	0.747 **																		
	M	0.092	0.116	0.086	0.665 **	0.678 **	0.647 **	0.827 **																	
	L	0.007	0.016	0.007	0.773 **	0.789 **	0.806 **	0.913 **	0.908 **																
*C. lanatus* W6 16791	E	0.891 **	0.878 **	0.891 **	0.051	0.022	0.088	0.187	0.099	0.138															
	M	0.949 **	0.939 **	0.954 **	0.007	−0.001	0.042	0.119	0.108	0.110	0.959 **														
	L	0.963 **	0.952 **	0.966 **	−0.018	−0.028	0.002	0.045	0.067	0.045	0.938 **	0.989 **													
*C. palaestinus* PI 235663	E	0.993 **	0.988 **	0.995 **	−0.063	−0.077	−0.067	−0.001	0.068	0.005	0.895 **	0.957 **	0.973 **												
	M	0.992 **	0.988 **	0.996 **	−0.081	−0.090	−0.083	−0.014	0.058	−0.009	0.881 **	0.946 **	0.958 **	0.996 **											
	L	0.992 **	0.990 **	0.996 **	−0.060	−0.071	−0.063	0.008	0.087	0.017	0.888 **	0.954 **	0.963 **	0.995 **	0.997 **										
*C. turkestanicus* PI 426180	E	−0.018	−0.014	−0.023	0.620 **	0.647 **	0.683 **	0.917 **	0.815 **	0.893 **	0.153	0.093	0.029	−0.022	−0.037	−0.021									
	M	0.004	0.014	0.004	0.683 **	0.706 **	0.699 **	0.796 **	0.895 **	0.933 **	0.076	0.072	0.038	0.006	−0.011	0.008	0.879 **								
	L	−0.007	−0.004	−0.005	0.735 **	0.753 **	0.749 **	0.894 **	0.840 **	0.905 **	0.117	0.109	0.041	0.000	−0.015	0.013	0.917 **	0.848 **							
*C. turkestanicus* PI 426181	E	−0.013	−0.011	−0.016	0.689 **	0.709 **	0.708 **	0.920 **	0.843 **	0.914 **	0.149	0.093	0.033	−0.012	−0.028	−0.011	0.935 **	0.882 **	0.907 **						
	M	0.054	0.068	0.051	0.695 **	0.709 **	0.678 **	0.825 **	0.954 **	0.922 **	0.100	0.098	0.063	0.048	0.032	0.055	0.862 **	0.949 **	0.877 **	0.922 **					
	L	0.025	0.040	0.025	0.682 **	0.687 **	0.714 **	0.854 **	0.945 **	0.955 **	0.107	0.100	0.043	0.021	0.008	0.033	0.889 **	0.946 **	0.904 **	0.915 **	0.972 **				
*C. turkestanicus* PI 426425	E	0.355 **	0.363 **	0.349 **	0.550 **	0.585 **	0.574 **	0.900 **	0.830 **	0.832 **	0.444 **	0.424 **	0.374 **	0.346 **	0.328 *	0.348 *	0.865 **	0.786 **	0.829 **	0.874 **	0.839 **	0.832 **			
	M	0.522 **	0.538 **	0.517 **	0.493 **	0.490 **	0.498 **	0.702 **	0.866 **	0.775 **	0.509 **	0.538 **	0.511 **	0.511 **	0.492 **	0.517 *	0.712 **	0.782 **	0.720 **	0.740 **	0.852 **	0.840 **	0.891 **		
	L	0.346 **	0.357 **	0.343 **	0.565 **	0.607 **	0.564 **	0.766 **	0.860 **	0.865 **	0.362 **	0.389 **	0.356 **	0.340 *	0.321 *	0.345 **	0.790 **	0.894 **	0.780 **	0.789 **	0.873 **	0.865 **	0.875 **		0.898 **

** Correlation significant at *p* < 0.01 (two-tailed). *Correlation significant at *p* < 0.05 (two-tailed). Sample name abbreviations: the first two letters indicate the first two letters of the species name, the number indicates the last digit of the PI number, and the letters E, M, and L indicate early, middle, and late stages of development, respectively. For example, “ti1E” indicates the sample PI 592391 (*C*. *tinctorius*) at the early stage of development.

**Table 2:** Details of safflower (*Carthamus*) wild and cultivated species samples used in this study.

Accession Number	Species	Flower Color at Middle Stage of Development	Spine Length	Type	Leaf Shape	Leaf Apices
PI 202728	*C. lanatus*	White	Long	Wild	Lanceolate	Attenuate
PI 235666	*C. lanatus*	Cream	Long	Wild	Lanceolate	Attenuate
W6 16791	*C. lanatus*	Yellow	Long	Wild	Lanceolate	Attenuate
PI 235663	*C. palaestinus*	Orange	Very short	Wild	Lanceolate	Acuminate
PI 426180	*C. turkestanicus*	Cream	Long	Wild	Lanceolate	Attenuate
PI 426181	*C. turkestanicus*	Cream	Long	Wild	Lanceolate	Attenuate
PI 426425	*C. turkestanicus*	Light-yellow	Long	Wild	Lanceolate	Attenuate
PI 592391	*C. tinctorius*	Orange	Very short	Cultivar	Lanceolate	Acuminate

## Supplementary Materials

The following are available online at https://www.mdpi.com/2218-1989/10/11/440/s1. Table S1: Relative peak areas of metabolites identified in florets of cultivated and safflower wild species, Table S2: Contents (mM) of metabolites with high VIP values in the florets of safflower.
