# Supplementary material for: Assessment of Metabolic Profiles in Florets of Carthamus Species Using Ultra-Performance Liquid Chromatography-Mass Spectrometry
Source: Metabolites. 2020 Oct 30;10(11):440. doi: 10.3390/metabo10110440 (PMC7693801; doi:10.3390/metabo10110440)
Supplement: Supplementary file 1 [file metabolites-10-00440-s001.pdf]

Assessment of metabolic profiles from florets of *Carthamus* species using ultra-performance liquid chromatography-mass spectrophotometry

Jiseon Kim[1,†], Awraris Derbie Assefa[2,†], Jaeeun Song [1], Vimalaj Mani[1], Soyoung Park [1], Seon-Kyeong Lee [1], Kijong Lee[1], Dong-Gwan Kim[3], and Bum-Soo Hahn[2,*]

1 Department of Agricultural Biotechnology, National Institute of Agricultural Sciences, Rural Development Administration, Jeonju 54874, Korea; jiseon07100@gmail.com (J.K.); icanje@korea.kr(J.S.); vimalraj08@gmail.com (V.M.); psy0203@korea.kr (S.P.); lsk220@korea.kr (S.-K.L.); leekjong@kroea.kr (K.L.)

2 National Agrobiodiversity Center, National Institute of Agricultural Sciences, Rural Development Administration, Jeonju 54874, Korea; awraris@korea.kr (A.D.A)

3 Department of Bio-Industry and Bio-Resource Engineering, Sejong University, Seoul, 05006, Korea; kimdg@sejong.ac.kr (D.-G.K.)

*Correspondence: bshahn@korea.kr; Tel.: +82-63-238-4930

† These authors contributed equally to this work

Abbreviations:

ti1E = PI 592391 (*C. tinctorius*) Early stage; ti1M = PI 592391 (*C. tinctorius*) Middle stage; ti1L = PI 592391 (*C. tinctorius*) Late stage; la8E = PI 202728 (*C. lanatus*) Early stage; la8M = PI 202728 (*C. lanatus*) Middle stage; la8L = PI 202728 (*C. lanatus*) Late stage; la6E = PI 235666 (*C. lanatus*) Early stage; la6M = PI 235666 (*C. lanatus*) Middle stage; la6L = PI 235666 (*C. lanatus*) Middle stage; la1E = W6 16791 (*C. lanatus*) Early stage; la1M = W6 16791 (*C. lanatus*) Middle stage; la1L W6 16791 (*C. lanatus*) Late stage; pa3E = PI 235663 (*C. palaestinus*) Early stage ; pa3M = PI 235663 (*C. palaestinus*) Middle stage; pa3L = PI 235663 (*C. palaestinus*) Late stage; tu0E = PI 426180 (*C. turkestanicus*) Early stage; tu0M = PI 426180 (*C. turkestanicus*) Middle stage; tu0L = PI 426180 (*C. turkestanicus*) Late stage; tu1E = PI 426181 (*C. turkestanicus*) Early stage; tu1M = PI 426181 (*C. turkestanicus*) Middle stage; tu1L = PI 426181 (*C. turkestanicus*) Late stage; tu5E = PI 426425 (*C. turkestanicus*) Early stage; tu5M = PI 426425 (*C. turkestanicus*) Middle stage; tu5L = PI 426425 (*C. turkestanicus*) Late stage

Table S1. Relative peak areas of metabolites identified in florets of cultivated and safflower wild species

S/No	Name	Mass	ti1E	ti1M	ti1L	la8E	la8M	la8L	la6E	la6M	la6L
1	1,5-Dicaffeoylquinic acid	516.12678				10.63527	14.13566	24.89657	23.90506	10.07133	15.9628
2	2'-Hydroxygenistein	286.04774				8.295862	14.66176	5.428298	9.929528	6.206962	5.355221
3	3'-*O*-Methylluteolin	300.06339	0.044034		0.02187	4.367496	7.698412	4.224443	7.368569	4.551709	7.320435
4	5-*O*-Caffeoylshikimic acid	336.08452		0.017001	0.152531						0.086937
5	Anhydrosafflor yellow B	1044.27468	8.74319	8.555967	10.51548						
6	Dihydrokaempferol	288.06339	0.370211	0.588973	0.085374						
7	Astragalin	448.10056	3.694385	2.381678	3.43947	2.007592	1.996224	2.931665	4.161253	3.043181	3.625632
8	Baimaside	626.1483	9.684891	8.939165	12.11722						
9	Caffeic acid	180.04225	0.602977	0.109797	0.143867	0.453771	0.23012	0.167607	4.157277	1.245387	1.892364
10	Caffeoylquinic acid	354.09508	0.98855	0.384338	1.438018	5.362128	3.362617	4.197394	8.00624	2.043434	3.962741
11	Eriodictyol	288.06339			0.04435						
12	Esculetin	178.02661			0.07479			0.110825	0.066373		0.199115
13	Ferulic acid	194.05791	0.121416	0.076032		0.157909	0.177255		0.376644	0.103227	0.360338
14	Glucoaurantio-obtusin	492.12677		0.061595	0.259917						
15	Isoquercetin	464.09548	0.74777	0.63955	1.376528	18.29982	20.75944	22.36358	9.29926	8.597489	9.085269
16	3-*O*-Methylquercetin	316.0583	0.098659			4.185999	6.463516	4.849368	11.8626	3.306829	2.510867
17	Kaempferin	432.10565	0.081962	0.024552	0.261181	1.609891	1.674311	2.460987	2.837157	2.211591	2.13535
18	Kaempferol	286.04774	1.335385	0.655222	0.296773	1.11751	2.22179	2.281991	9.095492	2.678143	1.941697
19	Kaempferol 3-*O*-β-*D*-glucosylgalactoside	610.15339	3.626354	3.695208	4.825598	2.491568	2.917092	4.041669	3.324219	5.344937	3.983958
20	Kaempferol 3-*O*-β-rutinoside	594.15847	9.752254	11.77993	9.691659	1.007646	1.462183	1.365986	8.542852	11.72285	6.364765
21	Malonylgenistin	518.10604						0.666046	0.102098	0.242851	0.320377
22	Myricitrin	464.09548				18.13296	20.5407	22.03154	9.223711	8.52977	9.124655
23	Narcissoside	624.16904	0.170793	0.167735	0.254671	0.450843	0.660151	0.530307	3.347893	4.209372	2.634027
24	Naringenin	272.06847	0.097705	0.369901	0.042008				0.072429	0.052438	
25	Prunin	434.1213		0.117728	0.649323						

26	Narirutin	578.16356							0.086683	0.112104	0.032889
27	Protocatechuic acid	154.02661				0.013177	0.056031	0.066637			0.060143
28	Quercetin	302.04265	0.250083	0.199696	0.047487	4.341684	6.77249	6.706237	12.55368	2.517849	5.557613
29	Quinic acid	192.06339	9.541625	8.312723	6.341498	3.004392	1.884496	1.220281	4.398483	2.531545	1.996263
30	Rutin	610.15339	1.39168	2.045068	2.634009	0.395694	0.622945	0.655205	11.40558	9.606239	10.75437
31	Safflomin A	612.16904	85.83412	76.73301	96.17824	0.341847	0.433196	0.427914	3.351074	3.626593	2.605451
32	Salicylic acid	138.03169	0.073136						0.089068	0.070509	0.431233
33	Scolymoside	594.15847	9.438958	11.54956	9.146443	0.992029	1.449578	1.353228	8.443017	11.51937	6.286109
34	Syringetin	346.06887					0.018936	0.040793	0.045574		
35	*p*-Coumaric acid	164.04734	0.217451	0.790948	0.672458		0.119614	0.059629	0.175017	0.373851	0.50437
36	Trifolin	448.10056				1.965891	2.068247	3.937579	4.119288		3.62063
37	Vitexin	432.10565									
38	Xanthorin	300.06339				4.274172	7.537761	4.147402	7.264636	4.456344	7.19969
39	α-Ketoglutaric acid	146.02152		0.679774	0.206013		0.204187		0.244877	0.428571	0.285189
40	6-Gingerol	294.18311			0.030815						
41	6-Hydroxydaidzein	270.05282				0.140882	0.10251	0.096151	0.291491	0.325027	0.202852
42	Acacetin	284.06847								0.550377	0.142019
43	Apigenin	270.05282				1.197766	2.972124	1.523791	1.948798	1.340055	1.535074
44	Apigenin 7-*O*-β-*D*-glucuronide	446.08491	0.324651	0.332431	0.368894	7.055908	8.35823	8.363029	14.07543	14.64676	10.52783
45	Cinnamic acid	148.05243								0.120538	0.037374
46	Herbacetin	302.04265	0.115357	0.033196							
47	Kaempferol 7-O-β-D-glucopyranoside	448.10056	0.942894	0.60132	0.79497	1.813893	1.195968	2.325741	1.634979	1.507958	1.558935
48	Luteolin 7-*O*- β-*D*-glucuronide	462.07983	0.407519	0.397967	0.470004	15.75652	20.20213	18.86678	23.38955	23.95479	20.38679
49	Luteolin 7-*O*-β-*D*-glucoside	448.10056	0.59439	0.579997	0.728516	1.923269	2.37189	5.325576	1.056769	2.802993	3.677955
50	Myricetin	318.03757					0.274045				
51	Pantothenic acid	241.09262	0.026859	0.023698	0.05587	0.368906	0.320913	0.31353	0.103811		0.035764
52	Phthalic acid	166.02661			0.47628	0.879941	0.990615	1.723539	0.688261	0.708072	0.853496

53	Pratensein	300.06339						1.122762		1.08522	1.381727
54	Quercetin 3-*O*-(6-*O*-malonyl-β-*D*-glucoside)	550.09587	0.033777	0.033718		3.07787	4.377166	6.853967	1.242246	3.012111	4.033636
55	Scutellarein	286.04774	0.0333	0.038325		0.403883	1.578243	0.658764	2.911176	1.033162	0.56756
56	Aconitic acid	174.01644	0.566481	0.7124	0.854962	1.334472	2.049256	1.427695	0.848963	2.517532	2.13098

Table S1 continued

S/No	Name	Mass	la1E	la1M	la1L	pa3E	pa3M	pa3L	tu0E	tu0M	tu0L
1	1,5-Dicaffeoylquinic acid	516.12678	7.628423	6.046094	2.549232				16.65229	12.54033	12.91707
2	2'-Hydroxygenistein	286.04774							5.486001	4.149949	6.164831
3	3'-*O*-Methylluteolin	300.06339	0.079547		0.14083			0.030911		7.76467	
4	5-*O*-Caffeoylshikimic acid	336.08452		0.01196			0.060382	0.140294		0.017521	0.036815
5	Anhydrosafflor yellow B	1044.27468			0.006211	12.76259	17.35375	18.57921			
6	Dihydrokaempferol	288.06339				0.737767	0.784908	0.048804			
7	Astragalin	448.10056	0.5	0.860394	0.891588	5.884745	2.982355	4.897516	2.46243	2.895847	3.48659
8	Baimaside	626.1483				11.26463	14.70059	10.35915		0.394688	
9	Caffeic acid	180.04225	2.720279	0.162945	0.06428	0.323999	0.258929	0.448022	2.455005	0.901826	1.336541
10	Caffeoylquinic acid	354.09508	8.436147	2.67935	1.129873	2.173363	1.759326	2.363569	4.925306	2.521461	2.377958
11	Eriodictyol	288.06339		0.030271	0.111947			0.219825			
12	Esculetin	178.02661			0.049737			0.090432			0.412867
13	Ferulic acid	194.05791	0.275721	0.220364	0.06024	0.063564			0.361526	0.19505	
14	Glucoaurantio-obtusin	492.12677	0.320289	0.447238	0.414007	0.217438	0.788937	0.911822			
15	Isoquercetin	464.09548	0.536004	0.746401	0.721975	1.730271	1.470664	6.914535	3.769031	6.805507	8.657835
16	3-*O*-Methylquercetin	316.0583	1.165607	1.094412		0.028544	0.030344	0.087948	6.444226	1.426995	8.114954
17	Kaempferin	432.10565	0.091343	0.126058	0.164209	0.056009		0.147332	1.936756	1.718643	2.194441
18	Kaempferol	286.04774	0.089702	0.098804	0.081246	0.314164	0.253434	0.827829	4.851105	1.454714	3.90787
19	Kaempferol 3-*O*-β-*D*-glucosylgalactoside	610.15339	2.119089	4.044454	4.239245	8.711621	5.531778	6.55368	4.018159	6.760082	4.371775
20	Kaempferol 3-*O*-β-rutinoside	594.15847				6.405853	7.165456	12.40902	4.686958	6.175118	7.189653

21	Malonylgenistin	518.10604							0.148851		0.265328
22	Myricitrin	464.09548	0.539235	0.843776					3.752259	6.734495	8.575512
23	Narcissoside	624.16904				0.139242	0.247573	0.44103	1.83639	2.151757	2.897457
24	Naringenin	272.06847	0.008001			0.034181	0.050308	0.121849	0.053404	0.04371	
25	Prunin	434.1213					0.131021	0.265639			
26	Narirutin	578.16356							0.067224	0.079262	0.035606
27	Protocatechuic acid	154.02661		0.014289	0.107453						0.045972
28	Quercetin	302.04265	2.251513	1.737722	0.50404	0.172583	0.171683	0.368307	6.604034	1.714703	7.915493
29	Quinic acid	192.06339	3.845369	0.998201	1.205767	6.907112	4.719519	4.459292	3.609446	1.9047	1.591084
30	Rutin	610.15339	2.165607	4.133467		1.48231	2.752732	6.452116	8.046471	3.794753	11.89685
31	Safflomin A	612.16904	28.19802	37.76693	28.65037	96.53826	95.27505	116.6579	1.696708	2.220265	2.53972
32	Salicylic acid	138.03169			0.060139				0.166696	0.161305	0.252638
33	Scolymoside	594.15847				6.240885	6.898834	2.858142	4.632883	6.078613	
34	Syringetin	346.06887	0.047287								0.027518
35	*p*-Coumaric acid	164.04734	0.056929	0.153736	0.117199	0.109978	0.636425	1.011454	0.383621	0.237554	0.291777
36	Trifolin	448.10056	0.497846	0.860288	0.881792	5.866994	2.970328	1.09379	2.462295	2.884815	3.492958
37	Vitexin	432.10565				0.081854					
38	Xanthorin	300.06339	0.079752	0.015083	0.140426			0.030911	7.050004	7.586122	6.116488
39	α-Ketoglutaric acid	146.02152		0.379922	0.177691	0.087431	0.384578	0.110764	0.21849	0.299017	0.098173
40	6-Gingerol	294.18311			0.026358			0.118675			
41	6-Hydroxydaidzein	270.05282				0.043176					
42	Acacetin	284.06847								0.714147	0.412076
43	Apigenin	270.05282					0.035961		3.002371	3.344999	2.165017
44	Apigenin 7-*O*-β-*D*-glucuronide	446.08491	0.090266	0.105419	0.085033	3.866395	0.727578	1.632659	9.519188	9.95819	10.51281
45	Cinnamic acid	148.05243								0.084407	0.11156
46	Herbacetin	302.04265				0.0707					
47	Kaempferol 7-O-β-D-glucopyranoside	448.10056	0.203662	0.32229	0.269441	2.05313	1.156603	1.248942	0.653413	0.940855	1.611956

48	Luteolin 7-*O*- β-*D*-glucuronide	462.07983	0.471433	0.536833	0.545849	1.270748	0.761524	0.634867	18.74206	22.141	20.36824
49	Luteolin 7-*O*-β-*D*-glucoside	448.10056	0.329829	0.443215	0.547869	1.076637	0.785213	0.7454	0.571339	0.881153	0.580765
50	Myricetin	318.03757	0.052929						0.119778		
51	Pantothenic acid	241.09262	0.120115	0.038209	0.090992	0.240885	0.060871	0.113845	0.120047	0.097108	0.03119
52	Phthalic acid	166.02661	0.221715	1.232007	1.047516	0.226553	0.708651	0.652484	0.207711		0.261563
53	Pratensein	300.06339	0.037337						5.481975	3.509131	2.722912
54	Quercetin 3-*O*-(6-*O*-malonyl-β-*D*-glucoside)	550.09587	0.340137	0.450095	0.547112			0.08781	3.003444	5.075925	4.174825
55	Scutellarein	286.04774		0.030747		0.055109	0.057757	0.056854	1.073754	0.541531	1.810673
56	Aconitic acid	174.01644	1.571136	1.860129	1.737831	1.956105	1.65816	1.197102	1.930092	2.499166	1.549854

Table S1 continued

S/No	Name	Mass	tu1E	tu1M	tu1L	tu5E	tu5M	tu5L
1	1,5-Dicaffeoylquinic acid	516.12678	17.39849	10.48626	14.78946	14.57613	6.232896	10.10014
2	2'-Hydroxygenistein	286.04774	7.469295	5.754437	3.873506	5.809176	1.809591	6.099066
3	3'-*O*-Methylluteolin	300.06339	5.21538	5.135376	4.394958	5.098439	1.960348	9.555538
4	5-*O*-Caffeoylshikimic acid	336.08452			0.025265			0.012422
5	Anhydrosafflor yellow B	1044.27468						
6	Dihydrokaempferol	288.06339						
7	Astragalin	448.10056	1.853747	2.910199	3.413793	3.264158	2.205854	4.176984
8	Baimaside	626.1483						
9	Caffeic acid	180.04225	1.79217	0.670377	0.857858	2.113263	0.297362	0.836926
10	Caffeoylquinic acid	354.09508	5.925615	3.072755	3.095197	4.332011	1.661265	2.062725
11	Eriodictyol	288.06339						0.051227
12	Esculetin	178.02661		0.043407	0.140207			0.509701
13	Ferulic acid	194.05791	0.258949	0.159452	0.086321	0.299494		
14	Glucoaurantio-obtusin	492.12677						
15	Isoquercetin	464.09548	5.79368	7.638568	8.168089	3.493606	3.503165	5.471153
16	3-*O*-Methylquercetin	316.0583	6.917785	2.699373	2.761124	9.510883	1.413278	3.565034

17	Kaempferin	432.10565	1.984955	1.825904	2.339649	1.874837	1.042538	3.396571
18	Kaempferol	286.04774	5.384955	2.801418	1.89695	5.484472	0.855624	1.997844
19	Kaempferol 3-*O*-β-*D*-glucosylgalactoside	610.15339	4.041219	5.98969	5.064869	3.585104	3.539626	5.01776
20	Kaempferol 3-*O*-β-rutinoside	594.15847	4.701566	8.67682	8.637021	8.028342	6.63635	8.607689
21	Malonylgenistin	518.10604		0.252944	0.369865	0.110027	0.189598	0.29191
22	Myricitrin	464.09548	5.722036	7.604007	8.092921	3.471215	3.460424	5.474233
23	Narcissoside	624.16904	1.840324	3.848515	3.489245	3.093898	2.506862	2.374756
24	Naringenin	272.06847				0.032831		
25	Prunin	434.1213						
26	Narirutin	578.16356	0.036409	0.11335	0.095482	0.080328	0.063098	
27	Protocatechuic acid	154.02661		0.039072	0.04518			0.114978
28	Quercetin	302.04265	5.856152	3.152422	3.323205	7.94269	1.093736	3.441125
29	Quinic acid	192.06339	3.252125	1.708453	1.453738	2.943108	1.718945	2.368751
30	Rutin	610.15339	9.584228	8.255111	12.4625	6.803017	5.105788	8.955703
31	Safflomin A	612.16904	2.213535	3.441509	2.758279	12.88225	10.86676	12.21404
32	Salicylic acid	138.03169		0.117333	0.333845	0.056736	0.062187	0.110615
33	Scolymoside	594.15847	4.647148	8.56552	8.52424	7.880109	6.635033	8.447387
34	Syringetin	346.06887	0.021477					
35	*p*-Coumaric acid	164.04734	0.056096	0.310351	0.229316	0.086174	0.098141	0.166051
36	Trifolin	448.10056	1.847483	2.884951	1.795778	3.246255	2.218312	4.193101
37	Vitexin	432.10565			0.023159			0.025562
38	Xanthorin	300.06339	5.089485	5.039306	4.311597	2.484002	1.907581	9.320193
39	α-Ketoglutaric acid	146.02152	0.278635	0.53998	0.136281			
40	6-Gingerol	294.18311			0.031695			
41	6-Hydroxydaidzein	270.05282						
42	Acacetin	284.06847		0.151309	0.250882		0.056616	
43	Apigenin	270.05282	1.525336	1.884014	1.62894	1.388851	0.878311	5.526948

44	Apigenin 7-*O*-β-*D*-glucuronide	446.08491	13.32148	13.79574	13.85763	12.66407	8.66167	11.42434
45	Cinnamic acid	148.05243	0.087808	0.158339	0.129851			
46	Herbacetin	302.04265						
47	Kaempferol 7-O-β-D-glucopyranoside	448.10056	4.491779	5.183176	1.963412	1.02949	0.495923	1.411816
48	Luteolin 7-*O*- β-*D*-glucuronide	462.07983	29.12808	28.31111	26.83072	20.59058	13.49213	17.58249
49	Luteolin 7-*O*-β-*D*-glucoside	448.10056	0.794911	1.053483	0.844315	0.683752	0.367904	1.548866
50	Myricetin	318.03757				0.715538		
51	Pantothenic acid	241.09262	0.133557	0.078437	0.062934			
52	Phthalic acid	166.02661	0.336801		1.161773			0.732574
53	Pratensein	300.06339		1.674477	2.163423			6.009547
54	Quercetin 3-*O*-(6-*O*-malonyl-β-*D*-glucoside)	550.09587	2.822092	6.108195	7.889894	0.958088	2.510964	6.58726
55	Scutellarein	286.04774	1.750503	1.685314	1.162627	3.023383	0.739353	1.672364
56	Aconitic acid	174.01644	2.243624	3.504716	2.030386	1.817318	1.750392	1.561647

Table S2. Contents (mM) of metabolites with high VIP values in the florets of safflower wild species.

No	**Mode**	**Metabolite**	**Formula**	**Mass**	**RT**	**ti1E**	**ti1M**	**ti1L**	**pa3E**	**pa3M**	**pa3L**
1	Neg	Quinic acid	$C_7H_{12}O_6$	192.0634	1.26	26.31±0.91	22.85±0.71	17.36±1.64	14.56±4.91	9.31±2.08	12.03±4.99
2	Pos	Pantothenic acid	$C_9H_{16}NO_5$-Na	241.0926	2.2	0.57±0.57	0.51±0.51	1.21±0.07	4.04±0.12	1.01±0.10	2.50±0.11
3	Neg	5-*O*-Caffeoylshikimic acid	$C_{16}H_{16}O_8$	336.0845	5.78	ND	0.04±0.04	0.86±0.37	ND	0.24±0.24	0.80±0.28
4	Neg	Kaempferol 3-*O*-β-*D*-glucosylgalactoside	$C_{27}H_{30}O_{16}$	610.1534	6.29	2.98±2.06	3.06±0.36	4.22±0.12	6.17±1.91	3.65±1.05	6.04±2.57
5	Neg	Rutin	$C_{27}H_{30}O_{16}$	610.1534	6.56	1.32±0.73	2.06±0.33	2.76±0.28	1.08±0.07	2.16±0.05	7.22±3.89
6	Neg	p-Coumaric acid	C_9H_8O3	164.0473	6.67	20.42±1.39	74.03±32.42	63.86±16.45	8.35±1.85	46.42±6.34	97.67±8.05
7	Neg	Myricitrin	$C_{21}H_{20}O_{12}$	464.0955	6.79	ND	ND	ND	ND	ND	ND
8	Neg	Scolymoside	$C_{27}H_{30}O_{15}$	594.1585	6.86	8.11±2.05	10.03±1.92	8.00±2.25	4.12±0.28	4.50±0.63	2.49±2.49
9	Neg	Isoquercetin	$C_{21}H_{20}O_{12}$	464.0955	6.93	2.18±1.10	1.75±0.98	4.84±0.48	4.74±0.60	3.81±0.69	28.32±23.76
10	pos	Luteolin 7-*O*-β-*D*-glucoside	$C_{21}H_{20}O_{11}$	448.1006	6.95	5.02±2.13	4.92±0.89	6.28±2.17	7.26±1.85	5.18±1.57	6.55±1.86
11	Neg	Safflomin A	$C_{27}H_{32}O_{16}$	612.169	6.98	77.82±17.01	70.03±3.32	88.64±4.14	69.78±2.52	67.68±6.26	109.14±4.66
12	Neg	Ferulic acid	$C_{10}H_{10}O_4$	194.0579	7.18	14.55±14.55	7.85±7.85	ND	4.00±0.40	ND	ND
13	Neg	Narcissoside	$C_{28}H_{32}O_{16}$	624.169	7.35	0.53±0.01	0.53±0.01	0.58±0.01	0.50±0.00	0.54±0.04	0.68±0.05
14	Neg	Kaempferol 3-*O*-β-rutinoside	$C_{27}H_{30}O_{15}$	594.1585	7.45	18.35±5	22.34±4.56	18.58±5.53	9.41±0.55	10.38±1.31	24.33±2.9
15	Neg	Astragalin	$C_{21}H_{20}O_{11}$	448.1006	7.57	9.63±5.59	6.28±2.56	9.14±2.76	12.18±0.45	6.12±0.31	13.2±0.23
16	Pos	Luteolin 7-O-glucuronide	$C_{21}H_{18}O_{12}$	462.0798	7.62	2.73±1.66	2.64±0.49	3.54±0.68	5.39±1.48	4.86±0.02	5.57±0.53
17	Neg	Trifolin	$C_{21}H_{20}O_{11}$	448.1006	7.72	ND	ND	ND	2.84±0.09	1.52±0.07	0.74±0.74
18	Neg	1,5-Dicaffeoylquinic acid	$C_{25}H_{24}O_{12}$	516.1268	7.73	ND	ND	ND	ND	ND	ND
19	pos	6-Hydroxydaidzein	$C_{15}H_{10}O_5$	270.0528	8.3	ND	ND	ND	0.12±0.12	ND	ND
20	Pos	Apigenin 7-O-β-D-glucuronide	$C_{21}H_{18}O_{11}$	446.0849	8.46	0.48±0.02	0.49±0.04	0.52±0.01	3.74±1.03	0.67±0.19	1.6±0.69
21	Neg	Kaempferin	$C_{21}H_{20}O_{10}$	432.1057	8.46	0.07±0.03	0.02±0.02	0.20±0.10	0.04±0.00	ND	0.12±0.09
22	Neg	Malonylgenistin	$C_{24}H_{22}O_{13}$	518.106	8.55	ND	ND	ND	ND	ND	ND
23	Neg	Anhydrosafflor yellow B	$C_{48}H_{52}O_{26}$	1044.275	9.03	31.75±1.81	31.17±14.25	39.49±10.45	37.29±5.53	50.68±5.32	73.13±2.32
24	Neg	2'-Hydroxygenistein	$C_{15}H_{10}O_6$	286.0477	9.45	ND	ND	ND	ND	ND	ND
25	Neg	Xanthorin	$C_{16}H_{12}O_6$	300.0634	11.14	ND	ND	ND	ND	ND	0.01±0.00
26	pos	Pratensein	$C_{16}H_{12}O_6$	300.0634	11.26	ND	ND	ND	ND	ND	ND
27	Neg	3'-*O*-Methylluteolin	$C_{16}H_{12}O_6$	300.0634	11.46	0.01±0.00	ND	0.01±0.00	ND	ND	0.01±0.00

"Pos" and "Neg" indicates the positive and negative mode of analysis of the mass spectrometer, respectively. ND (Not Detected): the amount of the metabolite was below detection limits of the method. ND = Not detected

Table S2 continued.

No	Metabolite	la8E	la8M	la8L	la6E	la6M	la6L	la1E	la1M	la1L
1	Quinic acid	6.28±0.84	3.30±0.02	1.69±0.57	8.56±0.19	4.13±0.23	3.41±0.62	8.99±0.66	1.21±0.03	1.91±0.05
2	Pantothenic acid	6.83±0.59	5.81±0.30	5.76±0.4	1.72±0.04	ND	0.63±0.63	2.37±0.08	0.74±0.07	1.82±0.05
3	5-*O*-Caffeoylshikimic acid	ND	ND	ND	ND	ND	0.40±0.04	ND	ND	ND
4	Kaempferol 3-*O*-β-*D*-glucosylgalactoside	1.59±0.52	1.89±0.20	2.87±0.08	1.98±0.09	3.36±0.66	2.67±0.22	1.38±0.04	2.99±0.12	3.34±0.1
5	Rutin	0.15±0.02	0.36±0.02	0.4±0.01	9.68±0.93	7.82±0.46	9.71±0.14	2.01±0.05	3.92±0.12	ND
6	p-Coumaric acid	ND	9.77±2.53	5.05±0.04	12.9±0.95	26.35±8.73	39.1±0.95	5.15±0.12	13.10±0.37	10.51±0.2
7	Myricitrin	110.44±10.39	122.45±0.23	133.43±1.01	48.35±1.85	42.85±1.29	51.03±1.32	0.92±0.06	1.94±0.19	ND
8	Scolymoside	0.48±0.18	0.81±0.12	0.75±0.04	5.56±0.37	7.42±2.67	4.34±0.27	ND	ND	ND
9	Isoquercetin	64.65±6.31	71.71±0.19	78.42±0.38	28.65±0.68	25.46±0.71	29.81±0.56	1.16±0.33	1.87±0.29	1.91±0.31
10	Luteolin 7-*O*-β-*D*-glucoside	14.41±0.44	17.37±1.98	39.62±1.2	6.99±0.14	17.98±2.22	26.04±0.19	2.59±0.26	3.35±0.15	4.36±0.73
11	Safflomin A	1.64±0.05	1.71±0.02	1.71±0.06	3.70±0.31	3.8±0.33	3.3±0.22	24.74±1.72	31.7±4.62	25.48±1.14
12	Ferulic acid	13.64±5.67	15.62±4.59	ND	36.69±0.42	8.04±0.04	37.47±0.92	31.14±0.74	22.53±2.70	4.95±4.95
13	Narcissoside	0.65±0.05	0.74±0.03	0.68±0	1.83±0.03	2.13±0.37	1.61±0.23	ND	ND	ND
14	Kaempferol 3-*O*-β-rutinoside	1.34±0.39	2.04±0.24	1.92±0.08	12.42±2.98	16.56±0.87	9.77±0.59	ND	ND	ND
15	Astragalin	4.61±4.61	4.53±0.53	6.64±6.64	8.48±0.50	6.01±0.13	7.86±0.02	1.31±0.21	2.11±0.02	2.28±0.07
16	Luteolin 7-O-glucuronide	237.40±20.82	302.07±17.69	285.08±7.81	318.45±32.08	314.42±17.7	293.92±20.92	3.08±0.37	3.6±0.69	3.96±0.56
17	Trifolin	1.08±0.08	1.11±0.11	2.14±0.94	2.02±0.09	ND	1.90±0.01	0.47±0.05	0.64±0.00	0.68±0.02
18	1,5-Dicaffeoylquinic acid	23.91±1.13	31.02±2.59	55.37±3.77	47.6±1.61	19.37±1.18	33.83±1.62	18.15±1.03	13.95±5.21	6.19±0.9
19	6-Hydroxydaidzein	0.38±0.04	0.25±0.05	0.24±0.04	0.62±0.03	0.66±0.01	0.48±0.04	ND	ND	ND
20	Apigenin 7-O-β-D-glucuronide	11.79±1.84	14.3±1.23	14.57±0.11	24.06±0.07	24.17±3.46	18.29±2.02	0.29±0.02	0.3±0.02	0.29±0.01
21	Kaempferin	1.01±0.10	1.03±0.05	1.53±0.07	1.58±0.02	1.19±0.19	1.26±0.05	0.07±0.02	0.09±0.03	0.12±0.01
22	Malonylgenistin	ND	ND	4.03±0.65	0.6±0.01	1.31±0.28	1.87±0.08	ND	ND	ND
23	Anhydrosafflor yellow B	ND	ND	ND	ND	ND	ND	ND	ND	0.02±0.00
24	2'-Hydroxygenistein	3.62±0.49	6.57±0.53	2.20±0.29	3.87±0.04	2.16±0.55	2.04±0.65	ND	ND	ND
25	Xanthorin	1.6±0.21	2.87±0.25	1.53±0.07	2.49±0.53	1.41±0.29	2.64±0.69	0.02±0.01	ND	0.04±0.00
26	Pratensein	ND	ND	1.46±0.04	ND	1.19±0.19	1.75±0.15	0.05±0.05	ND	ND
27	3'-*O*-Methylluteolin	1.35±0.18	2.46±0.22	1.29±0.06	2.11±0.04	1.19±0.25	2.24±0.06	0.02±0.01	ND	0.04±0.00

ND = Not detected

Table S2 continued.

No	Metabolite	**tu0E**	**tu0M**	**tu0L**	**tu1E**	**tu1M**	**tu1L**	**tu5E**	**tu5M**	**tu5L**
1	Quinic acid	9.78±1.01	4.29±0.3	3.34±0.25	6.66±0.19	2.64±0.17	2.14±0.81	6.41±1.06	3.30±0.24	4.99±2.06
2	Pantothenic acid	2.71±0.11	2.12±0.66	0.68±0.68	2.41±0.23	1.35±0.13	1.12±1.12	ND	ND	ND
3	5-*O*-Caffeoylshikimic acid	ND	0.05±0.05	0.11±0.09	ND	ND	0.07±0.00	ND	ND	0.01±0.00
4	Kaempferol 3-*O*-β-*D*-glucosylgalactoside	3.61±0.95	6.20±0.39	3.81±0.32	2.80±0.65	4.19±0.21	3.57±0.07	2.64±0.09	2.69±0.10	3.98±0.51
5	Rutin	9.33±1.15	4.24±0.24	13.37±0.44	8.88±0.58	7.26±1.54	11.41±0.66	6.7±0.21	5.13±0.73	9.04±1.50
6	p-Coumaric acid	38.23±2.04	22.94±0.53	28.04±1.75	4.56±4.56	23.7±9.94	18.08±5.04	7.54±0.82	8.81±0.53	14.56±0.60
7	Myricitrin	25.73±0.71	46.49±1.67	59.75±2.93	31.96±1.41	41.26±18.9	45.46±2.92	19.85±0.35	20.47±1.74	33.43±0.10
8	Scolymoside	4.10±0.29	5.27±0.73	ND	3.23±0.43	5.91±0.48	6.06±0.04	6.11±0.18	5.27±0.19	6.69±0.22
9	Isoquercetin	15.49±0.99	27.64±0.64	35.29±1.90	19.24±0.21	24.45±1.83	27.00±1.74	12.13±0.10	12.56±1.07	19.83±0.05
10	Luteolin 7-*O*-β-*D*-glucoside	5.15±2.08	7.69±0.20	5.04±0.17	5.74±0.09	7.28±0.86	5.99±0.04	5.28±0.08	2.93±0.29	12.25±7.05
11	Safflomin A	2.99±0.03	3.41±0.31	3.7±0.04	3.06±0.08	3.87±0.05	3.43±0.05	11.86±0.11	10.49±2.65	11.49±0.41
12	Ferulic acid	50.37±1.10	26.37±2.37	ND	25.84±0.6	15.81±0.81	7.25±7.25	33.71±7.12	ND	ND
13	Narcissoside	1.48±0.11	1.62±0.33	2.03±0.04	1.28±0.00	2.11±0.08	2.00±0.13	1.95±0.00	1.70±0.49	1.62±0.21
14	Kaempferol 3-*O*-β-rutinoside	9.23±0.66	11.83±1.67	13.79±0.98	7.33±0.93	13.19±1.07	13.53±0.05	13.72±0.42	11.63±4.26	14.99±0.89
15	Astragalin	6.88±1.49	7.79±0.98	9.33±0.30	4.13±0.13	6.22±0.84	7.49±0.38	7.80±0.11	5.47±0.12	10.12±0.13
16	Luteolin 7-O-glucuronide	350.85±48.11	402.63±35.02	367.76±13.17	440.72±22.91	407.6±8.19	397.20±72.08	329.18±14.69	216.08±10.92	283.3±53.65
17	Trifolin	1.69±0.33	1.88±0.22	2.23±0.06	0.99±0.99	1.53±0.2	0.95±0.95	1.88±0.04	1.39±0.02	2.41±0.68
18	1,5-Dicaffeoylquinic acid	45.35±1.69	32.97±0.71	33.86±8.72	37.9±3.48	21.83±8.61	31.67±1.56	34.03±2.09	15.02±1.51	23.99±0.8
19	6-Hydroxydaidzein	ND	ND	ND	ND	ND	ND	ND	ND	ND
20	Apigenin 7-O-β-D-glucuronide	21.94±2.66	22.18±1.13	23.57±2.04	25.05±1.63	24.72±3.41	25.70±3.44	25.59±1.17	16.81±1.86	23.13±5.29
21	Kaempferin	1.47±0.36	1.26±0.17	1.6±0.07	1.21±0.06	1.06±0.19	1.40±0.16	1.22±0.08	0.70±0.00	2.24±0.09
22	Malonylgenistin	1.14±0.71	ND	1.92±0.09	ND	1.47±0.67	2.18±0.03	0.74±0.11	1.28±0.24	1.91±0.28
23	Anhydrosafflor yellow B	ND	ND	ND	ND	ND	ND	ND	ND	ND
24	2'-Hydroxygenistein	2.82±0.61	1.94±0.04	3.08±0.07	3.11±0.23	2.17±0.17	1.37±0.07	2.52±0.20	0.51±0.02	2.72±0.17
25	Xanthorin	3.36±0.8	3.5±2.09	2.78±0.42	1.87±0.27	1.76±0.05	1.53±0.04	0.98±0.09	0.68±0.03	3.9±0.05
26	Pratensein	9.47±4.79	5.89±0.59	4.34±0.88	ND	1.93±0.65	2.69±0.29	ND	ND	9.03±1.60
27	3'-*O*-Methylluteolin	ND	3.01±0.81	ND	1.6±0.22	1.49±0.04	1.29±0.38	1.68±0.02	0.56±0.02	3.36±0.49

ND = Not detected
